# Factors influencing hepatitis C treatment initiation in a high-prevalence Brazilian state

**DOI:** 10.1590/0102-311XEN233025

**Published:** 2026-07-20

**Authors:** Vitória Machado Krüger, Ana Carolina Almeida da Silva, Marlise Antunes Grahl, Matheus dos Anjos Catasblancas, Maiara Lenise Lütz, João Pedro Stepan Wagner, Alexandre Lemos da Silva, Eduardo Viegas da Silva, Pedro Augusto Crespo da Silva

**Affiliations:** 1 Centro Estadual de Vigilância em Saúde, Secretaria da Saúde do Rio Grande do Sul, Porto Alegre, Brasil.; 2 Universidade Federal de Santa Maria, Santa Maria, Brasil.

**Keywords:** Hepatitis C, Liver Diseases, Time-to-Treatment, Unified Health System, Hepatite C, Hepatopatias, Tempo para o Tratamento, Sistema Único de Saúde, Hepatitis C, Hepatopatías, Tiempo de Tratamiento, Sistema Único de Salud

## Abstract

Hepatitis C virus infection remains a leading cause of chronic liver disease worldwide, affecting more than 70 million people, many of whom remain undiagnosed or untreated. In Brazil, Rio Grande do Sul State has the highest hepatitis C detection rate, underscoring the need for coordinated strategies to eliminate it by 2030. Underdiagnosis and barriers to treatment initiation persist despite the availability of highly effective antiviral therapies. This study aimed to estimate the proportion of diagnosed individuals who initiated treatment and to find factors associated with non-initiation of treatment. A retrospective cohort included all hepatitis C cases that had been diagnosed in 2022-2023 and reported in the Brazilian Information System for Notifiable Diseases (SINAN). Treatment initiation was verified in the Logistics Control System for Medications for Viral Hepatitis (SICLOM-HV). Probabilistic record linkage was performed to integrate both databases and to assess factors associated with non-initiation of treatment. Among 2,671 diagnosed individuals, 64.9% started treatment, with a median of 104 days between diagnosis and initiation. Multivariable analysis showed lower initiation among Black individuals than in White ones (adjusted OR = 1.41; 95%CI: 1.02-1.93) and among those aged 0-39 than in those aged 50-59 years (adjusted OR = 0.68; 95%CI: 0.49-0.96). Regional differences, HIV coinfection, and clinical variables also affected initiation. Treatment coverage in Rio Grande do Sul State remains below elimination targets. Persistent disparities by race, region, and coinfection highlight the need for targeted strategies to ensure equitable hepatitis C care and accelerate elimination efforts.

## Resumo

A infecção pelo vírus da hepatite C permanece como uma das principais causas de doença hepática crônica no mundo, afetando mais de 70 milhões de pessoas, muitas das quais permanecem sem diagnóstico ou tratamento. No Brasil, o Rio Grande do Sul apresenta a maior taxa de detecção de hepatite C, ressaltando a necessidade de estratégias coordenadas para alcançar a eliminação até 2030. Apesar da disponibilidade de terapias antivirais altamente eficazes, o subdiagnóstico e as barreiras para o início do tratamento persistem. Este estudo teve como objetivo estimar a proporção de indivíduos diagnosticados que iniciaram tratamento e identificar fatores associados à não iniciação. Uma coorte retrospectiva incluiu todos os casos de hepatite C diagnosticados em 2022-2023 e notificados no Sistema de Informação de Agravos de Notificação (SINAN). O início do tratamento foi verificado no Sistema de Controle Logístico de Medicamentos para Hepatites Virais (SICLOM-HV). Foi realizada vinculação probabilística de registros para integrar os bancos de dados e avaliar fatores associados à não iniciação do tratamento. Entre os 2.671 indivíduos diagnosticados, 64,9% iniciaram o tratamento, com mediana de 104 dias entre o diagnóstico e o início. A análise multivariada mostrou menor início entre indivíduos negros em comparação aos brancos (OR ajustada = 1,41; IC95%: 1,02-1,93) e entre aqueles com 0-39 anos em comparação a 50-59 anos (OR ajustada = 0,68; IC95%: 0,49-0,96). Diferenças regionais, coinfecção por HIV e variáveis clínicas também afetaram a iniciação. A cobertura de tratamento no Rio Grande do Sul permanece abaixo das metas de eliminação. Disparidades persistentes por raça, região e coinfecção destacam a necessidade de estratégias direcionadas para garantir cuidado equitativo e acelerar a eliminação da hepatite C.

## Resumen

La infección por el virus de la hepatitis C sigue siendo una de las principales causas de enfermedad hepática crónica en todo el mundo, afectando a más de 70 millones de personas, muchas de las cuales permanecen sin diagnóstico o sin tratamiento. En Brasil, el estado de Rio Grande do Sul tiene la tasa más alta de detección de hepatitis C, lo que subraya la necesidad de estrategias coordinadas para eliminar esta enfermedad para el 2030. A pesar de la disponibilidad de terapias antivirales altamente efectivas, persisten el infradiagnóstico y las barreras para iniciar el tratamiento. Este estudio buscó estimar la proporción de personas diagnosticadas que iniciaron el tratamiento e identificar los factores asociados con el fracaso en su inicio. Un estudio de cohorte retrospectivo incluyó todos los casos de hepatitis C diagnosticados entre el 2022 y el 2023 y notificados en el Sistema de Información de Agravios de Notificación (SINAN). El inicio del tratamiento se verificó en el Sistema de Control Logístico de Medicamentos contra la Hepatitis Viral (SICLOM-HV). La vinculación probabilística de registros trató de integrar ambas bases de datos y evaluar los factores asociados con la no iniciación del tratamiento. Entre los 2.671 individuos diagnosticados, el 64,9% inició el tratamiento, con una mediana de 104 días entre el diagnóstico y el inicio del tratamiento. El análisis multivariado mostró tasas de inicio más bajas entre las personas negras que entre las blancas (OR ajustado = 1,41; IC95%: 1,02-1,93) y entre los de 0 a 39 años que entre los de 50 a 59 años (OR ajustado = 0,68; IC95%: 0,49-0,96). Las diferencias regionales, la coinfección por VIH y las variables clínicas también influyeron en el inicio del tratamiento. La cobertura de tratamiento en Rio Grande do Sul sigue estando por debajo de las metas de eliminación. Las persistentes disparidades por raza, región y coinfección ponen de manifiesto la necesidad de estrategias que busquen asegurar un cuidado equitativo para la hepatitis C y acelerar los esfuerzos de eliminación.

## Introduction

Hepatitis C virus (HCV) infection has a high prevalence worldwide, standing out as one of the most important etiologies of chronic liver disease ^1^. Global estimates suggest that around 71 million people are infected by the virus and that 400,000 die annually due to disease complications. Notably, a substantial proportion of infected individuals remain undiagnosed and untreated ^2^ ^,^ ^3^. To address this challenge, the World Health Organization (WHO) has set the goal of eliminating hepatitis C as a public health threat by 2030 ^4^.

In Brazil, the state of Rio Grande do Sul has the highest detection rate in the country. In 2023, this rate was 28.1 cases per 100,000 inhabitants ^5^. While some individuals experience a self-limiting acute infection - often asymptomatic or accompanied by mild, nonspecific symptoms - about 85% progress to chronic infections, which can result in serious liver complications such as cirrhosis and hepatocellular carcinoma ^6^ ^,^ ^7^. Since only the chronic viral infection can be cured, hepatitis C requires focused strategies to identify asymptomatic carriers and expand access and adherence to treatment ^8^.

Diagnosis is essential to prevent complications and reduce transmission. All individuals diagnosed with HCV should receive treatment, regardless of disease stage ^9^ ^,^ ^10^. Intervention is based on direct-acting antivirals (DAAs), which achieves sustained virologic response rates above 95% and is associated with clinical improvement and reversal of liver damage ^11^. Since the introduction of DAAs in 2015, Brazil has expanded hepatitis C control via free treatment provided by the Brazilian Unified National Health System (SUS, acronym in Portuguese) and strategies such as rapid testing, decentralization of care to primary health services, and targeted micro-elimination initiatives for priority populations ^12^ ^,^ ^13^ ^,^ ^14^ ^,^ ^15^.

Despite these advances, progress toward hepatitis C elimination has varied across countries and regions. Structural and contextual barriers, such as health system constraints and social inequalities, continue to limit equitable access to diagnosis and treatment. Moreover, external disruptions, including interruptions to health services during the COVID-19 pandemic, sociopolitical instability and natural disasters, have further challenged the continuity of essential health services and hindered progress toward elimination targets ^16^ ^,^ ^17^. Consequently, many settings have increasingly adopted more realistic, context-specific approaches, including micro-elimination strategies focused on target populations ^18^.

In high-prevalence settings such as Rio Grande do Sul, understanding how these global challenges translate into regional barriers is essential to strengthen hepatitis C response strategies. Assessing gaps across the care continuum - including the identification of infected individuals, linkage to care, and timely treatment initiation - can support the identification of health inequities and inform the implementation of targeted interventions tailored to specific subgroups and territories.

## Methods

This retrospective cohort study was conducted using secondary data from SUS. The study population comprised all hepatitis C cases diagnosed in 2022 and 2023 in the state of Rio Grande do Sul and reported in the Brazilian Information System for Notifiable Diseases (SINAN, acronym in Portuguese). Eligible cases included new notifications with a reactive anti-HCV serology result and a detectable confirmatory viral load (HCV-RNA). Notifications of patients residing in other states and duplicate records in the system were excluded.

The cohort was monitored for 12 months to evaluate access to treatment based on records from the Logistics Control System for Medications for Viral Hepatitis (SICLOM-HV, acronym in Portuguese), including data recorded up to December 2024. Time to treatment was defined as the interval between the date of diagnosis and that of the first recorded treatment dispensation. A probabilistic record linkage algorithm was used to integrate SICLOM-HV treatment records with SINAN diagnosis information, and the selected variables were analyzed using Python, version 3.10.12 (http://www.python.org). Duplicates were removed from both systems after a manual evaluation of the accuracy of the linkage-generated information based on sensitivity and positive predictive values. Linkage spanned from 2022 to 2024.

To analyze the risk factors associated with treatment non-initiation, demographic and clinical-epidemiological variables were extracted from the notification form and compared between “treated” and “not treated” groups. Demographic data included age group (0-39, 40-49, 50-59, 60-69, and ≥ 70 years), sex (male, female), race/skin color (White, Black, Brown, other), education (up to incomplete 4th grade, complete 4th to incomplete 8th grade, complete elementary to incomplete high school, complete high school, and incomplete or complete higher education), and health macroregion of residence (Metropolitana, Centro-oeste, Missioneira, Norte, Serra, Sul, and Vales). The state of Rio Grande do Sul has seven health macroregions, large administrative regions that have been established to organize and decentralize health services and policies, enabling regionalized planning and the management of health care delivery ^16^. Clinical and epidemiological variables included HIV coinfection (yes, no), illicit drug use (yes, no), institutionalization (yes, no), and history of hemodialysis (yes, no).

Statistical analyses were performed on Stata, version 15.1 (https://www.stata.com). Categorical variables are shown as absolute (n) and relative (%) frequencies. To investigate the association between categorical variables and treatment groups (a binary variable), the Pearson’s chi-squared test was used, considering a statistical significance level of α = 0.05. Specifically, the variable “time between diagnosis and treatment initiation”, assessed as a continuous variable, is described using the mean and median due to its asymmetric distribution, which was confirmed by the Kolmogorov-Smirnov test (p < 0.001).

Logistic regression was used to assess the association between sociodemographic and clinical variables and the outcome “not treated”. Categories coded as “ignored” in the information systems were treated as missing data. Given the use of secondary data from routine health information systems, missing information in some variables was expected, reflecting the limitations of surveillance databases. A complete-case approach was adopted for the multivariable analysis. Variables with a high proportion of missing data (> 20%) - education level, institutionalization, illicit drug use, and history of hemodialysis - were excluded from the regression models. This decision was based on methodological considerations as including extensively missing variables could substantially reduce analytical sample size, compromise statistical power, and introduce instability or bias in the estimated associations.

Crude associations were first estimated using univariable logistic regression, shown as odds ratios (OR) with 95% confidence intervals (95%CI). Then, a hierarchical multivariable model with two levels was constructed based on epidemiological plausibility: level 1 (Model 1) included distal sociodemographic confounders (sex, age group, skin color) and level 2 (Model 2) added contextual variables (health macroregion, HIV coinfection). Model fit was assessed using the Hosmer-Lemeshow test and nested models (Model 1 vs. Model 2) were compared via the likelihood ratio test. Variables with p ≤ 0.20 in univariable analysis were considered for the multivariable model; some covariates were retained a priori for epidemiologic relevance. Variables were retained in the final model if statistically significant (p < 0.05) or if their inclusion/exclusion changed the OR of other variables by > 10%, indicating confounding. Multicollinearity was evaluated using the variance inflation factor (VIF), adopting the conventional criterion VIF > 10 as evidence of multicollinearity (in which VIF from 5 to 10 suggested moderate collinearity that warrants attention). Moreover, a tolerance < 0.10 (1/VIF) would indicate problematic collinearity. In our data, VIFs ranged from 1.01 to 2.46 in Model 1 (mean 1.61) and from 1.01 to 2.51 in Model 2 (mean 1.38), indicating no concerning multicollinearity.

This study was approved on 8 February 2024, by the Research Ethics Committee of the School of Public Health of Rio Grande do Sul (CAAE: 76496623.7.0000.5312).

## Results

### Data linkage

This study used records from the SINAN and SICLOM-HV databases for its linkage. The SINAN database comprised 4,000 records from 2022 to 2024, while the SICLOM-HV database contained 8,108 records from the same period. The automated linkage process initially found 2,961 potential matches in the databases. A manual review of all matched records found 250 false negatives and 38 false positives. The false negatives were individually verified and subsequently included, whereas the false positives were excluded. This validation confirmed 3,173 matches. Based on these results, linkage achieved a 92.1% sensitivity and a 98.7% positive predictive value. After completing the linkage and accuracy assessment, this study evaluated only SINAN cases diagnosed in 2022 and 2023, yielding a final analytical sample of 2,671 eligible cases. Of these, 1,734 cases were successfuly linked to corresponding SICLOM-HV records, while 937 cases had no treatment record.

### Sample characterization

The analysis in this study included 2,671 cases of hepatitis C reported in 2022 and 2023. Most patients were men or boys (n = 1,613; 60.38%) who self-identified as White (n = 1,903; 71.24%). The most prevalent age group was that of those aged 50-59 years (n = 770; 28.82%) and those aged 60-69 years (n = 656; 26.56%), with a mean age of 55 years (standard deviation - SD: ±13). Most cases referred to residents in the Metropolitana health macroregion (n = 1,616; 60.50%). Regarding educational level, the most common category was “completed 4th grade to incomplete 8th grade” (n = 607; 22.72%). Clinical and epidemiological data showed that 8.53% of individuals were institutionalized, 8.38% had HIV/AIDS coinfection, 17.67% reported illicit drug use, and 2.35% had undergone hemodialysis (Table 1). Supplementary Material (https://cadernos.ensp.fiocruz.br/static//arquivo/suppl-e00233025_5438.pdf) describes the analytical sample (including cases with missing data) in detail.

**Table 1:** Description of the analytical sample and bivariate analysis of factors associated with hepatitis C treatment initiation.

Variables	Total (n = 2,671)	Treated (n = 1,734)	Not treated (n = 937)	OR (95%CI)	p-value
	n (%)	n (%)	n (%)		
Sex					0.731
Male	1,613 (60.38)	1,043 (60.15)	570 (60.83)	1.00	
Female	1,058 (39.61)	691 (39.85)	367 (39.17)	0.97 (0.82-1.14)	
Race/Skin color *					0.004
White	1,903 (71.24)	1,286 (76.46)	617 (70.43)	1.00	
Black	333 (12.46)	193 (11.47)	140 (15.98)	1.51 (1.19-1.91)	
Brown	306 (11.45)	194 (11.53)	112 (12.79)	1.20 (0.93-1.54)	
Other **	16 (0.59)	9 (0.54)	7 (0.80)	1.62 (0.60-4.37)	
Age group (years)					0.001
0-39	314 (11.75)	190 (10.96)	124 (13.23)	1.00	
40-49	590 (22.08)	365 (21.05)	225 (24.01)	0.94 (0.71-1.25)	
50-59	770 (28.82)	517 (29.82)	253 (27.00)	0.74 (0.57-0.98)	
60-69	656 (26.56)	459 (26.47)	197 (21.02)	0.65 (0.49-0.87)	
≥ 70	341 (12.76)	203 (11.71)	138 (14.73)	1.04 (0.76-1.42)	
Education level *					0.467
Up to incomplete 4th grade	229 (8.57)	148 (12.81)	81 (14.78)	1.00	
Complete 4th to incomplete 8th grade	607 (22.72)	413 (35.76)	194 (35.40)	0.85 (0.62-1.18)	
Complete elementary to incomplete high school	434 (16.24)	288 (24.94)	146 (26.64)	0.92 (0.66-1.29)	
Complete high school	330 (12.35)	236 (20.43)	94 (17.15)	0.72 (0.50-1.04)	
Incomplete or complete higher education	103 (3.85)	70 (6.06)	33 (6.02)	0.86 (0.52-1.41)	
Macroregion of residence					< 0.001
Metropolitana	1,616 (60.50)	1,038 (59.86)	578 (61.69)	1.00	
Centro-oeste	197 (7.37)	132 (7.61)	65 (6.94)	0.88 (0.64-1.21)	
Missioneira	100 (3.74)	68 (3.92)	32 (3.42)	0.84 (0.54-1.30)	
Norte	145 (5.42)	104 (6.00)	41 (4.38)	0.70 (0.48-1.03)	
Serra	193 (7.22)	146 (8.42)	47 (5.02)	0.57 (0.40-0.81)	
Sul	299 (11.19)	160 (9.23)	139 (14.83)	1.56 (1.21-2.00)	
Vales	121 (4.53)	86 (4.96)	35 (3.74)	0.73 (0.48-1.09)	
Institutionalization *					0.426
No	1,700 (63.64)	1,141 (88.59)	559 (87.34)	1.00	
Yes	228 (8.53)	147 (11.41)	81 (12.66)	1.12 (0.84-1.50)	
HIV/AIDS coinfection					< 0.001
No	1,946 (72.85)	1,334 (92.00)	612 (85.00)	1.00	
Yes	224 (8.38)	116 (8.00)	108 (15.00)	2.02 (1.53-2.68)	
Illicit drug use *					0.004
No	1,300 (48.67)	922 (79.69)	378 (73.40)	1.00	
Yes	472 (17.67)	335 (20.31)	137 (26.60)	1.42 (1.11-1.81)	
History of hemodialysis *					0.050
No	1,622 (60.72)	1,117 (96.88)	505 (94.92)	1.00	
Yes	63 (2.35)	36 (3.12)	27 (5.08)	1.65 (0.99-2.76)	

95%CI: 95% confidence interval; OR: odds ratio.

* Missing data categories were suppressed from the table and excluded from the bivariate analysis;

** Yellow and Indigenous.

### Treatment initiation

**Figure 1:**
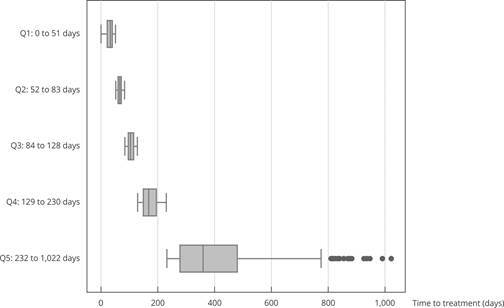
Time interval (in days) between hepatitis C diagnosis and treatment initiation.

Of the analyzed cases, 1,734 (64.91%) had a recorded treatment initiation. The median time from diagnosis to treatment initiation totaled 104 days, with an interquartile range (IQR) of 139 days, indicating that 50% of the central values fall into an interquartile range of 139 days. The distribution of this interval, divided into quintiles, showed that 20.36% of patients started treatment within 51 days. The last quintile (from 232 to 1,022 days) comprised 19.95% of the patients (Figure 1).

**Figure 2:**
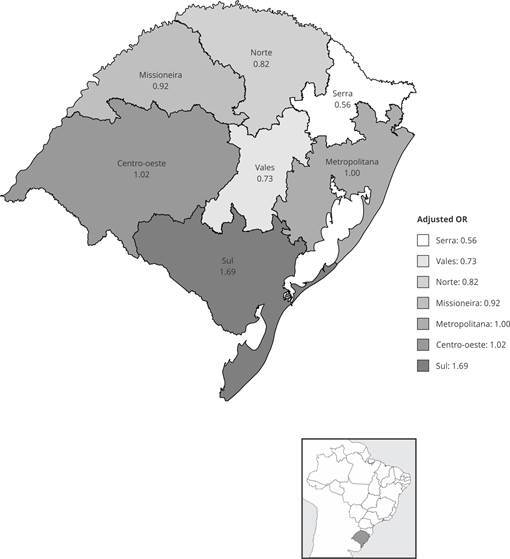
Geographic distribution of the seven macroregions of Rio Grande do Sul State, Brazil, according to adjusted odds ratios (OR) for hepatitis C treatment non-initiation.

In the bivariate analysis (Table 1), no statistically significant associations were observed between treatment initiation and sex, education level, institutionalization, or history of hemodialysis. In contrast, we found significant associations between race/skin color (OR = 1.51; 95%CI: 1.19-1.91), age group (OR = 0.65; 95%CI: 0.49-0.87), macroregion of residence (OR = 1.56; 95%CI: 1.21-2.00), HIV/AIDS coinfection (OR = 2.02; 95%CI: 1.53-2.68), and illicit drug use (OR = 1.42; 95%CI: 1.11-1.81). After adjusting for potential confounders in the logistic regression model restricted to complete cases, associations remained consistent with the bivariate findings. This analysis excluded education level, institutionalization, illicit drug use, and history of hemodialysis due to a high proportion of missing data (> 20%). In this context, Black individuals were less likely to initiate treatment than white ones (adjusted OR = 1.41; 95%CI: 1.02-1.93), whereas Brown ones showed a borderline association with lower treatment initiation (adjusted OR = 1.32; 95%CI: 0.98-1.79). Individuals aged 0-39 years had a lower probability of initiating treatment than those aged 50-59 years (adjusted OR = 0.68; 95%CI: 0.49-0.96) and 60-69 years (adjusted OR = 0.69; 95%CI: 0.49-0.98). Regarding macroregion of residence, individuals living in the Sul macroregion were less likely to initiate treatment (adjusted OR = 1.69; 95%CI: 1.27-2.26) than those in the Metropolitana macroregion (Figure 2). Finally, those with an HIV/AIDS coinfection were associated with a lower likelihood of initiating hepatitis C treatment than those without it (adjusted OR = 1.82; 95%CI: 1.29-2.57) (Table 2).

**Table 2:** Multivariate analysis of factors associated with non-initiation of hepatitis C treatment in complete cases (n = 1,762).

Varibles	OR	95%CI	Adjusted OR	95%CI
Sex				
Male	1.00			
Female	1.00	0.82-1.23	0.98	0.80-1.21
Race/Skin color				
White	1.00			
Black	1.39	1.01-1.90	1.41	1.02-1.93
Brown	1.32	0.98-1.78	1.32	0.98-1.79
Other *	1.68	0.37-7.56	1.63	0.36-7.38
Age group (years)				
0-39	1.00			
40-49	0.86	0.61-1.22	0.84	0.59-1.19
50-59	0.70	0.50-0.98	0.68	0.49-0.96
60-69	0.69	0.49-0.98	0.69	0.49-0.98
≥ 70	0.98	0.65-1.46	0.99	0.66-1.48
Macroregion of residence				
Metropolitana	1.00			
Centro-oeste	1.01	0.70-1.46	1.02	0.71-1.48
Missioneira	0.95	0.60-1.51	0.92	0.57-1.47
Norte	0.77	0.51-1.15	0.82	0.54-1.24
Serra	0.55	0.36-0.82	0.56	0.37-0.84
Sul	1.67	1.25-2.21	1.69	1.27-2.26
Vales	0.70	0.44-1.13	0.73	0.46-1.18
HIV/AIDS coinfection				
No	1.00			
Yes	1.92	1.38-2.68	1.82	1.29-2.57

95%CI: 95% confidence interval; OR: odds ratio.

* Yellow and Indigenous.

## Discussion

Despite significant progress in the Brazilian response to hepatitis C as a public health concern, this study findings show critical gaps in the care continuum, particularly regarding access to treatment. This is the first study in Brazil to evaluate the time to treatment initiation in a cohort of patients diagnosed with hepatitis C using probabilistic record linkage across SUS health information systems. Results showed that in the state of Rio Grande do Sul, the treatment initiation rate was 64.9%, below the 80% target set by the WHO to eliminate hepatitis C by 2030. Notably, this value is also below the national rates observed in 2023 (78%) and 2024 (73%) ^19^ ^,^ ^20^. This highlights a challenge for the state in meeting the targets for disease elimination. However, the proportion of patients treated in Rio Grande do Sul still exceeds the averages in high-income countries. Nguyen et al.’s ^21^ systematic review and meta-analysis of 146 studies involving over 1.7 million patients reported 64.2%, 45.4%, 54.4% treatment rates in South America, North America, and Europe, respectively. In the United States, Karmarkar et al. ^22^ observed even lower rates: only 33% of eligible patients initiated treatment with DAAs. Despite regional challenges, the SUS has ensured relatively broader access to treatment than those in many developed countries, primarily due to the universal and free provision of DAAs by SUS.

In Brazil, access to hepatitis C treatment varies substantially across states, reflecting heterogeneity in health system organization, service availability, and population profiles. Although the treatment initiation rate observed in Rio Grande do Sul lies below national averages, the state shows no worse treatment indicators of access than other Brazilian states ^19^. However, the implications of these gaps are particularly relevant in Rio Grande do Sul as it consistently reports the highest hepatitis C detection rate in the country and a high prevalence of HIV infection and other transmissible diseases. In this high-burden epidemiological context, even moderate losses along the care continuum translate into a large absolute number of untreated individuals, amplifying the public health impact ^5^ ^,^ ^23^. Barriers to treatment initiation likely reflect the influence of individual factors and structural determinants, including service centralization, territorial inequalities in access to specialized care, and limitations in the integration between surveillance, diagnosis, and treatment services.

Although this study did not assess the structural and organizational characteristics of health services, the literature indicates that these factors condition access to hepatitis C treatment, even under favorable economic indicators ^15^ ^,^ ^24^ ^,^ ^25^. Favorable macroeconomic conditions may coexist with organizational inefficiencies, fragmented service delivery, and persistent social inequalities, which limit individuals’ real opportunities to obtain timely treatment. When care coordination is inefficient, the entire population may experience reduced access to treatment. However, socially vulnerable subgroups (such as individuals with lower levels of empowerment or those belonging to historically marginalized groups) tend to be disproportionately affected. Moreover, these barriers often manifest themselves as problems in linkage to care and as prolonged delays between diagnosis and treatment initiation ^26^ ^,^ ^27^.

Consistent with this interpretation, our findings evince substantial delays in hepatitis C treatment initiation. Although the median time to treatment totaled 104 days, some patients only began treatment more than one year after their diagnosis. International studies have reported the wide variation in treatment delays; in a U.S. cohort, the median time to initiation was approximately 300 days, with fewer than 10% of patients starting treatment within the first year after diagnosis ^28^. Similarly, despite the high DAA national coverage in Brazil, available evidence remains limited, indicating substantial delays along the care continuum often exceeding one year between diagnosis and treatment or clinical follow-up ^29^ ^,^ ^30^. According to the Brazilian Clinical Protocols and Therapeutic Guidelines for Hepatitis C, treatment should begin immediately after diagnosis confirmation without the need to wait for clinical disease progression ^10^ ^,^ ^31^. Delays in treatment initiation, especially among vulnerable populations, are associated with a higher risk of loss to follow-up and worse clinical outcomes, such as progression of liver disease and lower cure rates ^32^.

The multivariate analysis in this study found significant demographic and clinical factors associated with treatment initiation. Black individuals were less likely to initiate treatment than white ones, as in previous studies that showed lower access to care in Black and Hispanic patients due to structural and institutional barriers ^33^. For instance, Kanwal et al. ^34^ reported that Black patients were 21% less likely to receive DAAs than white ones (OR = 0.79; 95%CI: 0.75-0.84). Racial and ethnic disparities in health care access are well documented across various conditions, including hepatitis C. Although non-White populations represent a minority in Rio Grande do Sul; a pattern fails to reflect the Brazilian population in which Black and Brown individuals constitute the majority. National evidence indicates that, even within a universal health system, these populations experience persistent disadvantages in accessing specialized and continuous care, largely driven by structural racism, socioeconomic inequalities, lower health literacy, and educational inequities. Therefore, the racial disparities in Rio Grande do Sul may be even more pronounced in other Brazilian states with higher proportions of non-White populations, highlighting the need to address structural and social determinants of access in strategies to improve hepatitis C treatment coverage ^25^ ^,^ ^35^.

Age group configured another relevant factor: individuals aged from 30 to 39 were less likely to initiate treatment than those aged 50 to 69. This finding may reflect lower risk perception, less frequent engagement with the health care system, and reduced prioritization of treatment in younger age groups. Previous studies have also reported that younger adults are more likely to be lost to follow-up and have lower adherence to treatment ^36^ ^,^ ^37^. Data from the 2019 *Brazilian National Health Survey* ^38^ indicate that patterns of health-service utilization vary by age group, in which younger adults generally report lower frequencies of service use and engagement than older ones. These age-related disparities suggest that care models that prioritize older age groups or advanced disease stages may less effectively reach younger individuals, underscoring the importance of strengthening health education and engagement strategies tailored to improve risk perception and continuity of care among younger adults.

Moreover, this study observed territorial disparities: residents of the Sul macroregion were less likely to begin treatment, whereas those from the Serra macroregion showed higher uptake. These regional inequalities may be linked to variations in the organization of local health systems, the availability of specialized services, and logistical constraints. A study in a municipality of the Sul macroregion found limited access to health services and significant sociodemographic vulnerabilities ^27^. International evidence indicates that decentralization and care integration strategies, particularly those focused on strengthening primary health care, are effective in expanding access to treatment ^15^. Such territorial disparities indicate that access to hepatitis C treatment follows the local organization of health services, reinforcing the role of place of residence as a determinant of health care access. Differences in regional capacity, service decentralization, and integration between primary and specialized care may exacerbate inequities, particularly in socially vulnerable areas.

Among the clinical factors, HIV coinfection was significantly associated with lower treatment initiation. Barriers in this population include drug interactions, adjustments in antiretroviral therapy, and poor adherence to follow-up care. Jatt et al. ^39^ reported that fewer than half of HIV-HCV coinfected individuals began treatment with DAAs, even among those with well-controlled HIV ^39^ ^,^ ^40^. These challenges are compounded by behavioral and social vulnerabilities, especially illicit drug use. Previous studies consistently show that people who inject drugs face stigma, logistical barriers, and low adherence to health services, which compromise access to and continuity of treatment ^41^ ^,^ ^42^ ^,^ ^43^. In Brazil, evidence indicates that people who use illicit drugs face additional structural barriers to accessing hepatitis treatment, including severe substance dependence, poor adherence to follow-up, stigma, and limited availability of user-friendly health services, all of which hinder consistent engagement in care ^22^ ^,^ ^44^.

This study has some limitations. Its use of secondary data from routine health information systems may suffer from underreporting, inconsistencies, and incomplete records, which may have affected the accuracy of some variables. A high proportion of missing information, particularly for education level, illicit drug use, institutionalization, and history of hemodialysis, limited the inclusion of these variables in the multivariable analysis in this study and may have reduced its ability to fully capture social and clinical vulnerabilities associated with treatment initiation. Moreover, probabilistic record linkage between information systems is subject to linkage losses due to data quality issues, such as incomplete or inconsistent identifiers, which can lead to misclassification of treatment status. Furthermore, the inability to exclude deaths during follow-up represents a potential source of overestimation of the proportion of untreated individuals. Finally, we applied no time-to-event methods to model the interval from diagnosis to treatment initiation due to data-structure and validation constraints. Thus, this research descriptively summarized time to treatment.

Despite these limitations, this study provides a relevant contribution to understanding access to hepatitis C treatment in Rio Grande do Sul. The findings show that, although the state has implemented a structured response, treatment coverage remains insufficient to ensure effective population-level management of hepatitis C. Overall, the racial, regional, and age-related inequalities in this study highlight the multifaceted nature of barriers to hepatitis C treatment initiation. These dimensions, rather than operating in isolation, interact to produce cumulative disadvantages in access to care. Addressing such inequities requires integrated, equity-oriented strategies that consider social vulnerability, the territorial organization of health services, and life-course differences in health care engagement. Key public health interventions include decentralization of care, micro-elimination approaches tailored to priority populations and territories, capacity-building of primary healthcare providers, and the integration of health information systems to address gaps. Strengthening the continuum of care by active surveillance and a patient-centered approach focused on vulnerable populations will be critical to accelerating progress toward the elimination of hepatitis C as a public health threat (even if achieving the 2030 target may be challenging in real-world settings).

## Data Availability

The research data are available upon request to the corresponding author.
