# Supplementary material for: Factors influencing hepatitis C treatment initiation in a high-prevalence Brazilian state
Source: Cad Saude Publica. 2026 Jul 20;42:e00233025. doi: 10.1590/0102-311XEN233025 (PMC13384362; doi:10.1590/0102-311XEN233025)
Supplement: Supplementary Material [file 1678-4464-csp-42-EN233025-s.pdf]

# SUPPLEMENTARY MATERIAL

Complete analytical sample, including missing data cases (n = 2,671).

| Variables | Total<br>n (%) |
|---|---|
| Sex | |
| Male | 1,613 (60.38) |
| Female | 1,058 (39.61) |
| Race/Skin color | |
| White | 1,903 (71.24) |
| Black | 333 (12.46) |
| Brown | 306 (11.45) |
| Other | 16 (0.59) |
| Missing | 113 (4.23) |
| Age group (years) | |
| 0-39 | 314 (11.75) |
| 40-49 | 590 (22.08) |
| 50-59 | 770 (28.82) |
| 60-69 | 656 (26.56) |
| ≥ 70 | 341 (12.76) |
| Education level | |
| Up to incomplete 4th grade | 229 (8.57) |
| Complete 4th to incomplete 8th grade | 607 (22.72) |
| Complete elementary to incomplete high school | 434 (16.24) |
| Complete high school | 330 (12.35) |
| Incomplete or complete higher education | 103 (3.85) |
| Missing | 923 (34.55) |
| Macroregion of residence | |
| Metropolitana | 1,616 (60.50) |
| Centro-oeste | 197 (7.37) |
| Missioneira | 100 (3.74) |
| Norte | 145 (5.42) |
| Serra | 193 (7.22) |
| Sul | 299 (11.19) |
| Vales | 121 (4.53) |
| Institutionalization | |
| No | 1,700 (63.64) |
| Yes | 228 (8.53) |
| Missing | 743 (27.81) |
| HIV/AIDS coinfection | |
| No | 1,946 (72.85) |
| Yes | 224 (8.38) |
| Missing | 501 (18.75) |
| Illicit drug use | |
| No | 1,300 (48.67) |
| Yes | 472 (17.67) |
| Missing | 899 (33.65) |
| History of hemodialysis | |
| No | 1,622 (60.72) |
| Yes | 63 (2.35) |
| Missing | 986 (36.91) |
